# Prediction of Clinically Significant Depressive Symptoms at 2-Year Follow-Up in Older Adults: Machine Learning Study Using the English Longitudinal Study of Ageing

**DOI:** 10.2196/84744

**Published:** 2026-07-02

**Authors:** Bahar Khorram, Ramin Nilforooshan, Payam Barnaghi, Samaneh Kouchaki

**Affiliations:** 1 Centre for Vision, Speech and Signal Processing (CVSSP), Department of Electrical and Electronic Engineering, Faculty of Engineering and Physical Sciences, University of Surrey, Stag Hill Campus, Guildford, GU2 7XH, United Kingdom, +44 (0)1483 683435; 2 Surrey and Borders Partnership NHS Foundation Trust, Leatherhead, United Kingdom; 3 School of Psychology, University of Surrey, Guildford, United Kingdom; 4 UK Dementia Research Institute, Care Research and Technology Centre, London, United Kingdom; 5 Department of Brain Sciences, Imperial College London, London, United Kingdom; 6 Department of Data Research, Innovation and Virtual Environments, Great Ormond Street Hospital NHS Foundation Trust, London, United Kingdom

**Keywords:** depressive symptoms, aged, machine learning, predictive analytics, English Longitudinal Study of Ageing, depression screening

## Abstract

**Background:**

Depression in older adults is often underdiagnosed due to atypical symptom presentation and generational stigma, leading to delayed intervention. Early identification of individuals at risk of developing elevated depressive symptoms is therefore critical, but traditional approaches show limited predictive accuracy. To date, no study has applied machine learning (ML) models to predict clinically significant depressive symptoms at 2-year follow-up in older adults in the United Kingdom using data from the English Longitudinal Study of Ageing (ELSA). Moreover, the impact of encoding strategies for categorical health care variables has not been examined.

**Objective:**

This study aimed to develop and evaluate ML models to predict the clinically significant depressive symptoms at 2-year follow-up in older adults using ELSA data. We further compared ordinal and one-hot encoding strategies across different ML architectures and identified key predictors of depressive symptoms at follow-up.

**Methods:**

Data were drawn from 4 consecutive waves of ELSA, including participants aged ≥50 years without significant depressive symptoms at the baseline wave (waves 6‐9). Clinically significant depressive symptoms were defined as 8-item Center for Epidemiologic Studies Depression Scale (CES-D 8) scores of ≥4 at the subsequent wave (waves 7‐10). Over 120 features spanning sociodemographic, psychological, and health-related domains were analyzed. Eight ML models were applied, including tree-based ensembles, deep learning architectures for tabular data, distance-based methods, probabilistic methods, and linear methods. Model performance was assessed using the area under the receiver operating characteristic curve (AUROC) and *F*_1_-score. Model interpretability was examined using Shapley additive explanations (SHAP). Sensitivity analyses assessed the robustness of results across alternative CES-D 8 thresholds (≥3, ≥4, and ≥5) and encoding strategies.

**Results:**

Across waves, the best-performing models achieved mean AUROC scores of 0.72‐0.73, with a peak of 0.75 in the highest-performing wave. Ordinal encoding consistently outperformed one-hot encoding across all ML models, yielding improvements in AUROCs and *F*_1_-scores, with the greatest increase in tree-based methods. SHAP consistently identified loneliness, sleep disturbances, and low social engagement as strong predictors of elevated depressive symptoms at follow-up. Sensitivity analyses across CES-D 8 thresholds demonstrated robust feature importance, with AUROCs ranging from 0.67 to 0.82. Traditional ML models (random forest, extreme gradient boosting, and support vector machines) generally achieved higher performance than the deep learning models for this task.

**Conclusions:**

Our findings demonstrate the feasibility of predicting clinically significant depressive symptoms at 2-year follow-up in UK older adults, with moderate accuracy. Ordinal encoding demonstrates superior performance for health care datasets with inherently ordered categorical features. The identification of consistent risk factors highlights opportunities for developing targeted clinical screening tools and preventive interventions. This study provides new evidence on depressive symptom prediction in the UK context, leveraging longitudinal data from ELSA, and contributes to advancing digital mental health research for aging populations.

## Introduction

Depression is one of the most widespread mental health disorders, characterized by low mood, loss of interest in activities, reduced energy, and cognitive difficulties persisting for at least 2 weeks. With 280 million people affected worldwide, it is a major contributor to global disability and disease burden [1]. In the United Kingdom, depression is among the most prevalent mental disorders [2]. Older adults are particularly vulnerable due to age-related health decline, cognitive impairment, reduced social interactions, and changes in family dynamics [3,4]. They also have the highest suicide rates of any age group [5].

Demographic shifts have exacerbated this issue. Over the past 40 years in the United Kingdom, the population aged 50 years and older has increased by 47%, and those aged 65 years and older by 52% [6]. Within this significant increase, depression often remains undiagnosed, leading to a reduced quality of life and increased mortality rates [7]. Therefore, early identification of individuals at risk for developing elevated depressive symptoms is critical for timely intervention, but this requires tools tailored to the risk factors most relevant to older adults.

The risk profile for depression in older adults differs from that of younger populations. While emotional symptoms such as stress are highlighted in younger cohorts, older adults with depression often experience cognitive difficulties, physical complaints, and reduced interest in activities [8,9]. Factors such as loss of independence, poor sleep, chronic diseases, and functional limitations are associated with the depression in older adults but do not necessarily predict depressive symptoms at follow-up [10].

Machine learning’s (ML’s) ability to analyze complex, multidimensional data and uncover subtle, nonlinear interactions has made it a powerful tool for predicting depressive symptoms. Prior studies have applied ML in a variety of contexts, ranging from social media monitoring to structured cohort studies. For instance, social media–based approaches have detected depressive language and emotional distress from user-generated content on platforms such as Twitter (X Corp), Facebook (Meta Platforms), and Reddit (Reddit, Inc), though their applicability to older adults is limited due to lower engagement with these platforms [11-13].

Beyond social media, many studies have focused on predicting depressive symptoms through questionnaires and interviews across different cohorts, including military cohorts [14,15], student populations [16], and occupational cohorts [17,18]. For example, in occupational cohorts, elastic net models have identified key predictors such as gender, anxiety disorders, and adverse life events [18]. Notable longitudinal studies have examined the risk of depression in older adults from China and Canada, incorporating variables such as emotional instability, low life satisfaction, perceived health, and nutrition risk, achieving areas under the receiver operating characteristic curves (AUROCs) ranging from 0.62 to 0.79 [7,19,20]. Overall, these studies show the feasibility of predicting depressive symptoms in older adults; despite this difference in cohorts, predictors, and modeling approaches limit the generalizability of these findings to UK older adult populations.

Recent ML approaches for depressive symptoms prediction in older adults have yielded promising results; however, several methodological limitations remain. First, most studies have been conducted outside the United Kingdom in populations with different health care systems and cultural contexts [7,19,20], limiting their applicability to UK clinical settings. Second, while tree-based methods have shown strong performance [18-20], few studies have systematically compared results across diverse algorithmic paradigms (eg, deep learning vs traditional ML), making it unclear which approaches are optimal. Third, categorical feature encoding strategies, which are particularly important for handling ordinal variables such as self-rated health and mobility limitations, have not been systematically evaluated, with most studies relying on one-hot encoding. Finally, deep learning architectures specifically designed for tabular data (eg, TabTransformer and TabNet) remain largely unexplored for depressive symptoms prediction in older adults, despite their potential advantages for handling mixed feature types. The English Longitudinal Study of Ageing (ELSA) offers comprehensive longitudinal data that can address these gaps. Existing ELSA-based research has examined associations between depression and factors such as frailty, biomarkers, and social conditions [21-23], yet no study has systematically applied ML approaches to predict future depressive symptoms at follow-up.

Compared with conventional statistical methods that rely on linearity and predefined interaction structures, ML approaches can capture nonlinear, complex interactions among heterogeneous features in high-dimensional data. We applied 8 ML models across diverse learning paradigms, including tree-based ensembles, deep learning architectures specifically designed for tabular data, distance-based methods, probabilistic methods, and linear methods. This systematic model selection ensures our findings on encoding strategies are algorithmically robust and generalizable rather than constrained to specific model structures. The objective of this study was to apply these diverse models to predict clinically significant depressive symptoms at 2-year follow-up in older adults using data from ELSA and to identify key risk factors contributing to depressive symptoms at follow-up. The 2-year prediction window was chosen to align with the biannual wave structure of ELSA. This timeframe also provides sufficient time for preventive interventions while minimizing uncertainty from longer-term changes in health status and life circumstances that complicate prediction accuracy.

This study makes 3 key contributions. First, this study provides a systematic ML-based approach to predicting depressive symptoms at follow-up among older adults in the United Kingdom using ELSA data, addressing a gap in UK-specific risk prediction models. Second, through a comprehensive comparison of 8 diverse ML algorithms (tree-based, deep learning, distance-based, probabilistic, and linear models), we demonstrate that ordinal encoding consistently outperforms one-hot encoding for health care datasets with naturally ordered categorical features, with average improvements of 0.013 in AUROC and 0.037 in *F*_1_-score. Third, we integrate multithreshold sensitivity analyses of the 8-item Center for Epidemiologic Studies Depression Scale (CES-D 8 score ≥3, ≥4, and ≥5) with individual-level Shapley additive explanations (SHAP) interpretability to identify robust risk factors that remain stable across depression severity levels, enabling targeted clinical interventions.

The best-performing models achieved AUROC values between 0.72 and 0.75 for 2-year depressive symptoms at follow-up. In this setting, traditional ML approaches generally achieved higher performance than the deep learning models in the ELSA dataset. SHAP analysis highlighted loneliness, sleep disturbances, and low social engagement as the robust predictors across all depression thresholds.

## Methods

### Study Design and Participants

ELSA is a continuous cohort study that collects detailed information on people in England aged 50 years or older. These data, which include lifestyle, health, psychological, and sociodemographic factors, were collected using computer-assisted interviews and self-reported questionnaires at 2-year intervals, or “waves” [24]. ELSA enables longitudinal tracking of health, economic, and social circumstances, with periodic refreshment of the panel by recruiting new participants aged 50 years and older during specific waves (3, 4, 6, 7, 9, and 10) to maintain representativeness. A large-scale longitudinal study like ELSA serves as a solid basis for investigating the underlying reasons for depression in older adults.

For this study, data from waves 6 (2012‐2013), 7 (2014‐2015), 8 (2016‐2017), and 9 (2018‐2019) were analyzed to predict depressive symptoms at follow-up in waves (7, 8, 9, and 10). Four of the most recent waves were chosen to ensure that the results accurately reflect contemporary aging and mental health patterns while providing sufficient longitudinal data to support robust analysis. Each wave pair was modeled independently rather than using cumulative training across all waves. This approach allowed us to examine whether predictive performance and risk factor associations remained consistent across different time periods while maximizing sample size for each prediction task. Restricting analyses to participants observed across all waves would substantially reduce the number of eligible cases due to attrition and missingness. To ensure a focus on incident depressive symptoms at follow-up rather than ongoing symptoms and eliminate any interfering factors, participants with severe psychiatric conditions, such as schizophrenia, psychosis, and bipolar disorder, as well as those expressing significant emotional or mood instability, were excluded. These exclusions were based on self-reported doctor-diagnosed conditions and relevant baseline ELSA mental health items.

The ELSA dataset demonstrates robust psychometric reliability across multiple domains, including sociodemographic, physical health, psychological, lifestyle, and social engagement. In the core member cohort, sample sizes ranged from 5362 to 6891 participants across waves 6‐9 [25], providing sufficient statistical power for reliable model training. The cohort shows demographic diversity across gender, age, marital status, and cultural background as summarized for wave 6 in Table 1. It also provides insight into sample distribution based on the depressive symptom status of the participants before analyzing model performance. Tables S1, S2, and S3 in Multimedia Appendix 1 present the corresponding characteristics for waves 7, 8, and 9 in Multimedia Appendix 1. Because of the high percentage of missing data for the education-level variable in the waves analyzed, this variable was removed from the tables.

Depressive symptoms were assessed using the 8-item Center for Epidemiological Studies Depression Scale (CES-D 8). This scale measures depression and demonstrates psychometric properties comparable to the original 20-item version. The CES-D 8 assesses both cognitive/affective symptoms (eg, felt depressed or was happy) and somatic symptoms (eg, restless sleep). A summary score (possible range: 0‐8) was calculated by adding responses, and this score was used to define the target variable for supervised model training.

**Table 1.** Sociodemographic characteristics, including gender, age group, marital status, and cultural background of English Longitudinal Study of Ageing (ELSA) participants at wave 6 (2012‐2013; n=8799), divided by depressive symptom status. Clinically significant depressive symptoms were defined using the 8-item Center for Epidemiologic Studies Depression Scale (CES-D 8) with a cutoff score of ≥4.

Variable	All participants (n=8799)	Nondepressed (n=6842)	Depressed (n=1957)
Sex, n (%)			
Male	3987 (45)	3312 (48)	675 (34)
Female	4812 (55)	3530 (52)	1282 (66)
Age (years), n (%)			
≥65	4851 (55)	3682 (54)	1169 (60)
<65	3948 (45)	3160 (46)	788 (40)
Marital status, n (%)			
Single (never married)	525 (6)	414 (6.1)	111 (5.7)
Married/civil partnership	5973 (68)	4893 (71.5)	1080 (55.2)
Previously married (separated/divorced/widowed)	2299 (26)	1533 (22.4)	766 (39.1)
Not available	2 (∼0)	2 (∼0)	0 (0)
Cultural background, n (%)			
English	6871 (78.1)	5358 (78.3)	1513 (77.3)
Irish/Scottish/Welsh	443 (5)	352 (5.1)	91 (4.7)
Other European	44 (0.5)	30 (0.4)	14 (0.7)
Other cultural backgrounds	172 (2)	138 (2)	34 (1.7)
Not available	1269 (14.4)	964 (14.1)	305 (15.6)

For the binary classification task, ${\text{y}}_{\text{i}}\text{∈\{0, 1\}}$ denote the depressive symptom status for participant i, where ${\text{y}}_{\text{i}}\text{=1}$ if CES_D_8≥4  (Class 1, depression) and ${\text{y}}_{\text{i}}\text{=0}$ if CES_D_8≤3 (Class 0, no depression) [25,26]. This threshold has been validated in ELSA and older adult populations, reflecting clinically meaningful levels of depressive symptoms. The CES-D 8 demonstrates strong internal consistency (Cronbach α=0.90‐0.92 across ELSA waves) and longitudinal measurement invariance, ensuring reliable depression classification across waves [26].

This criterion was applied at each wave to examine the emergence of new depression cases over the following 2 years. For each wave pair, this baseline exclusion criterion was used independently. Therefore, individuals who were identified as depressed at the baseline of a particular wave were excluded from that related analysis, even if they were not classified as depressed at earlier waves, ensuring that the learning task focused on incident depressive symptoms at follow-up rather than persistent or recurrent symptoms.

Participants who did not provide responses to any of the 8 CES-D items were excluded from the analysis, as a complete CES-D score was required to determine depressive symptom status, thereby minimizing label noise arising from missing symptom data. To assess the robustness of the depression labels, sensitivity analyses were conducted using alternative CES-D 8 thresholds (≥3 and ≥5), which showed consistent performance patterns and stable feature importance rankings across models, supporting the reliability of the outcome definition.

### Data Preprocessing and Feature Engineering

The ELSA dataset provides a rich set of variables collected across multiple domains and waves. For this analysis, data from 4 consecutive waves were used to predict the incidence of depression within the subsequent 2 years. A number of psychological variables were included in the analysis, including the Life Satisfaction Scale [27], which measures overall life contentment; the Quality of Life Questionnaire, which assesses autonomy, control, and self-realization; and the Loneliness Scale [28], which measures feelings of loneliness and perceived social isolation. In addition to psychological measures, nonpsychological variables were incorporated to provide a broader range of participants’ lives, spanning socioeconomic status, environmental exposures, health status, lifestyle behaviors, and cognitive function. To have a depressive symptom prediction model that is accurate and consistent, careful selection and efficient preprocessing of variables are important. In addition to excluding participants with missing CES-D variables, missing predictor values were imputed. Mean imputation was used for continuous variables and mode imputation for categorical variables, applied separately within each wave.

To address the high dimensionality of the ELSA dataset, which contains over 6100 variables per wave, we applied systematic feature reduction and selection approaches. We developed a consistent feature set that included over 120 variables from all waves to develop depressive symptom prediction models. First, a variance threshold of 0.01 was applied to remove features with low variability, as they provide limited discriminative information. Features were subsequently selected based on well-established associations with depressive symptoms reported in prior epidemiological and clinical studies, supported by domain knowledge and clinical expertise. Features with known associations with mental health, such as sleep quality (heslpf), and self-rated health (hehelf), were prioritized, whereas administrative variables, technical survey metadata, and variables with limited relevance to mental health were excluded. While the total number of available features varied slightly between waves due to differences in data collection, we ensured consistency by prioritizing common features across waves. Additionally, wave-specific variables were incorporated, such as sleep-related variables available in waves 6 and 8, fruit and vegetable intake in waves 6‐8, cognitive measures in waves 7‐9, and sexual activity variables in wave 6. These variables were included only where available, resulting in slightly different feature sets across wave pairs. A complete list of all features is provided in Tables S4 and S5 in Multimedia Appendix 1. While predictive modeling was performed across all waves, feature importance and clustering analyses were conducted on one representative wave.

### ML Methods

To maintain a balance between interpretability, computational efficiency, and predictive accuracy among traditional and state-of-the-art models, the following ML algorithms were used for the prediction of depression.

- Logistic regression (LR): a baseline method for binary classification that models linear relationships between predictors and the outcome. This approach is valued for its simplicity and interpretable coefficients.
- Random forest (RF): an ensemble method that combines multiple decision trees. It improves classification performance by using bootstrap aggregating (bagging) and randomization in feature selection during tree construction to reduce overfitting and enhance robustness.
- Support vector machine (SVM): a supervised ML method that identifies an optimal hyperplane to separate classes in high-dimensional space with minimal error. Its capabilities make it a widely used option for both classification and regression tasks.
- Extreme gradient boosting (XGBoost): an ensemble gradient-boosting framework that iteratively refines predictions by correcting the residuals of previous models. It then aggregates results from all models to make the final prediction, making it highly effective as a predictive model. Given the nature of our dataset and XGBoost’s ability to handle missing data and identify complex interactions between features, it is a strong choice for this study.
- K-nearest neighbors (KNN): a nonparametric algorithm that classifies samples based on the label of the most frequent samples among its KNNs in the feature space. The idea behind the approach is that samples of the same class are usually grouped near each other in the feature space. Feature normalization was applied to optimize the distance metric.
- TabNet: a deep learning architecture designed for tabular data. TabNet enables sparse feature selection while maintaining interpretability by using a sequential attention mechanism. All these features make it an appropriate choice for tasks involving the classification of tabular data, such as depressive symptom prediction [29].
- TabTransformer: an attention-based model designed to emphasize categorical features. Initially, categorical features are mapped into contextual embeddings. After being concatenated with continuous features, they are subsequently processed through a multilayer perceptron for classification or regression tasks [30]. This approach leverages the rich categorical features of the dataset.
- Multinomial naive Bayes (MNB): a probabilistic classifier that assumes feature independence. Three approaches were tested: (1) using standard naive Bayes for the entire dataset, (2) applying MNB to the entire dataset, and (3) combining standard naive Bayes for numerical features and MNB for categorical features. The second strategy outperformed the other methods, indicating its effectiveness for this study. Continuous variables were discretized into 10 categories to adapt them for MNB. For instance, age ranging from 50 years to 90+ years was divided into 10 equal-width bins (50‐54, 54‐58, etc).

For each pair of waves, participants who were present in both the baseline wave and the target wave were identified. The matched participants were divided into training, validation, and test sets (70%, 15%, 15%). Internal validation was used for hyperparameter tuning. To ensure robustness, this procedure was performed 5 times using different random seeds. For each run, a new train-validation-test split was generated, and model performance was evaluated on the corresponding held-out test set. The classification threshold was selected based on the value that maximized the *F*_1_-score $\text{F}\text{1= }\frac{\text{2}\text{PR}}{\text{(}\text{P}\text{+}\text{R}\text{)}}$. on the validation set. This approach was adopted to ensure a balanced trade-off between precision and recall, which is particularly important given the class imbalance in the depression dataset.

Additional hyperparameters were optimized using cross-validation and included RF (n_estimators=300, max_depth=7, min_samples_split=5, min_samples_leaf=2, class_weight=“balanced_subsample”), XGBoost (n_estimators=200, max_depth=5, learning_rate=0.1), SVM (C=1.0, kernel=“rbf”), LR (C=1.0, penalty=“l2”), and KNN (n_neighbors=5). TabNet and TabTransformer used learning_rate=0.001 and batch_size=128. MNB used α=1.0.

### Feature Importance

To evaluate the importance of the features for depressive symptom prediction in our study, SHAP was used for XGBoost and RF. SHAP offered a clear way to understand how individual variables affect model predictions. Applying SHAP to our best-performing models highlighted the most significant features that played a crucial role in predictions. This approach enhanced model interpretability and facilitated a deeper understanding of the complex multidimensional risk factors associated with depression in older adults.

To investigate feature contribution variation at the individual level, SHAP force plots were performed for 2 representative cases, one participant who developed clinically significant depressive symptoms in the consecutive wave and one who remained nondepressed in both waves. To highlight a distinct risk profile, the cases were chosen based on high model confidence (predicted probabilities >0.80 for depressed cases and <0.10 for nondepressed cases).

### Sensitivity Analysis

To evaluate the robustness of results to key methodological choices, we performed sensitivity analyses examining CES-D 8 threshold selection and categorical feature encoding strategies.

#### Threshold Selection

To assess the influence of CES-D 8 threshold selection on depressive symptoms prediction, we conducted sensitivity analyses using different depression cutoffs (≥3, ≥4, and ≥5 symptoms) for both XGBoost and RF models. Increasing the CES-D 8 threshold makes the outcome definition stricter, shifting from a larger group with milder symptoms to a smaller group with more severe depressive symptomatology.

For each threshold, depressive symptom status at wave 7 (outcome) was redefined while preserving identical inclusion criteria, feature engineering, and model hyperparameters. Both models were retrained and evaluated on data having a depression label corresponding to each threshold. Feature importance was assessed using SHAP values, and the consistency of highly ranked predictors across thresholds was examined to evaluate the robustness of key risk factors under increasingly conservative definitions of depression.

#### Categorical Encoding

To validate our categorical encoding strategy, we performed a sensitivity analysis comparing 2 encoding approaches for categorical variables with natural ordering. For a categorical feature ${\text{x}}_{\text{j}}$ with k ordered levels, ordinal encoding maps categories to integer values*,* ${\text{x}}_{\text{j}}\text{∈\{1,2,…,}\text{k}\text{\}}$, preserving their natural sequence (eg, self-rated health: excellent=1, very good=2, good=3, fair=4, and poor=5). By contrast, one-hot encoding is represented ${\text{x}}_{\text{j}}$ as a binary indicator vector ${\text{e}}_{\text{j}}\text{=[}{\text{e}}_{\text{j}\text{1}}\text{,}{\text{e}}_{\text{j}\text{2}}\text{,…,}{\text{e}}_{\text{jk}}\text{]∈\{0,1}{\text{\}}}^{\text{k}}$, where ${\text{e}}_{\text{ji}}\text{=1 }$ if ${\text{x}}_{\text{j}}$ belongs to the i^th^ category and 0  otherwise. Ordinal variables such as self-rated health and mobility limitations were encoded as sequential integers under the ordinal approach, whereas one-hot encoding transformed these variables into binary indicators. This comparison was performed across all 6 ML algorithms using identical train-test splits, resampling strategies, and hyperparameters. Model performance was evaluated using AUROC and *F*_1_-score for the positive class (depression).

### Clustering

Different subset groupings and preprocessing strategies were examined to identify clustering patterns associated with depression. Three-group and 6-group feature sets were chosen for clustering analysis. The 3-group configuration divided features into 3 main categories, including demographic, physical health, and social engagement categories aligned with known risk factors. The 6-group approach organized these into demographic, physical health, social engagement, psychological, lifestyle, and cognition features to examine whether more detailed subgroups would reveal additional depression-related patterns. These configurations were chosen to balance interpretability with sufficient detail while ensuring adequate samples within each cluster. The 3-group approach was chosen because it produced more visually distinct groupings in t-distributed stochastic neighbor embedding (t-SNE) visualizations compared to the 6-group approach, which showed overlapping patterns. This suggests that aggregating features into broader categories captures more distinct patterns and provides clearer clinical interpretability.

Two approaches were used to represent the features. To enhance cluster separation, the first strategy applied ordinal encoding to categorical features and discretization to numerical features. In the second method, the TabTransformer model was used to encode categorical features into dense vectors, which were then combined with continuous features. K-Means clustering was applied iteratively in both strategies to determine the optimal number of clusters based on the Silhouette score, and t-SNE was used to visualize the results. It facilitated the qualitative evaluation of the methods to identify patterns related to depression. Findings from these analyses are discussed in the Results section.

### Ethical Considerations

This study used the publicly available, deidentified ELSA dataset. Ethical approval for each wave of ELSA was obtained from the South Central–Berkshire Research Ethics Committee (formerly the NRES Committee South Central–Berkshire), and all participants provided informed consent at the time of data collection. No new participant data were collected by the authors. For questions regarding the dataset, contact ELSA. No artificial intelligence tools were used for data analysis or interpretation in this study.

## Results

### Overview

This section presents the results of applying multiple ML models to 4 independent ELSA wave pairs to predict clinically significant depressive symptoms at 2-year follow-up in older adults. Across waves, the best-performing models had AUROC values between 0.72 and 0.75. Notably, traditional ML techniques—including RF, XGBoost, SVM, and MNB—generally achieved higher performance than the deep learning models designed for tabular data. Moreover, ordinal encoding consistently improved model performance for every algorithm compared to one-hot encoding. Robustness was assessed through sensitivity analyses examining alternative CES-D 8 thresholds and encoding strategies, which demonstrated stable performance patterns. Finally, feature importance analyses identified social engagement, sleep quality, and self-rated health as reliable and influential predictors of depressive symptoms at follow-up.

### Comparative Model Performance Across Waves

Models’ performance in predicting depressive symptoms varied across the 4 waves, and no single model consistently outperformed others in every wave. Table 2 reports wave-specific analytic sample sizes and incident case rates, which provide essential context for interpreting model performance in the presence of class imbalance. Table 3 presents the mean performance metrics across waves, focusing on clinically relevant Class 1. AUROC values in Table 3 are reported with 95% CIs to quantify uncertainty in model discrimination across waves. Detailed wave-specific results, as well as the mean performance metrics for Class 0, are provided in Multimedia Appendix 1.

**Table 2.** Wave-specific analytic sample size and incident depressive symptoms at follow-up.

Outcome wave	Baseline sample size (n)	Incident cases n (%)
Wave 7	5735	764 (13.3)
Wave 8	5128	828 (16.1)
Wave 9	4582	821 (17.9)
Wave 10	3144	650 (20.7)

**Table 3.** Comparative performance metrics of machine learning models for predicting depressive symptoms at follow-up among older adults (aged ≥50 years) using English Longitudinal Study of Ageing (ELSA), waves 6‐10. Clinically significant depressive symptoms were defined using 8-item Center for Epidemiologic Studies Depression Scale (CES-D 8) with a cutoff score ≥4.

Model	Precision (C1)^a^, mean (SD)	Recall (C1), mean (SD)	Specificity (C0)^b^, mean (SD)	*F*_1_ (C1), mean (SD)	Accuracy, mean (SD)	Macro *F*_1_, mean (SD)	AUROC^c^ (95% CI)
RF^d^	0.32 (0.015)	0.52 (0.022)	0.80 (0.010)	0.40 (0.018)	0.76 (0.009)	0.62 (0.012)	0.72 (0.70‐0.74)
TabNet	0.27 (0.017)	0.45 (0.025)	0.78 (0.011)	0.34 (0.020)	0.73 (0.010)	0.58 (0.013)	0.67 (0.65‐0.69)
XGBoost^e^	0.30 (0.014)	0.57 (0.023)	0.75 (0.012)	0.39 (0.017)	0.72 (0.010)	0.60 (0.011)	0.72 (0.70‐0.74)
KNN^f^	0.26 (0.018)	0.28 (0.020)	0.85 (0.008)	0.26 (0.016)	0.76 (0.007)	0.56 (0.012)	0.60 (0.58‐0.62)
LR^g^	0.30 (0.016)	0.44 (0.021)	0.82 (0.009)	0.35 (0.018)	0.76 (0.008)	0.60 (0.011)	0.71 (0.69‐0.73)
SVM^h^	0.30 (0.015)	0.57 (0.022)	0.76 (0.011)	0.40 (0.017)	0.73 (0.009)	0.61 (0.012)	0.72 (0.70‐0.74)
MNB^i^	0.32 (0.014)	0.56 (0.021)	0.78 (0.010)	0.40 (0.016)	0.75 (0.009)	0.62 (0.011)	0.73 (0.71‐0.75)
TabTransformer	0.29 (0.016)	0.60 (0.024)	0.73 (0.012)	0.39 (0.018)	0.71 (0.010)	0.60 (0.012)	0.71 (0.69‐0.73)

a C1: Class 1, indicates depressive symptoms at follow-up.

b C0: Class 0, indicates no depression.

c AUROC: area under the receiver operating characteristic curve.

d RF: random forest.

e XGBoost: extreme gradient boosting.

f KNN: k-nearest neighbors.

g LR: logistic regression.

h SVM: support vector machine.

i MNB: multinomial naive Bayes.

Overall, RF, XGBoost, MNB, and SVM were the best-performing models. These models achieved the highest balanced results, with mean AUROC scores between 0.72 and 0.73 and mean macro-average *F*_1_-scores ranging from 0.60 to 0.62. Despite their effectiveness in detecting participants without depression (*F*_1_-score=0.82‐0.85), their performance in identifying depressive symptoms at follow-up (*F*_1_-score=0.40) was more limited, highlighting challenges in predicting the minority class. Table 4 presents confusion matrices and predictive values for RF and XGBoost models trained on wave 6 predictors and evaluated at wave 7.

**Table 4.** Confusion matrices and predictive values for random forest (RF) and extreme gradient boosting (XGBoost) models predicting incident depressive symptoms at wave 7 using wave 6 predictors, evaluated on the held-out test set (n=1147; 12.4% incident cases).

Model	TN^a^	FP^b^	FN^c^	TP^d^	PPV^e^	NPV^f^
RF^g^	848	157	68	74	0.32	0.93
XGBoost^h^	816	189	61	81	0.30	0.93

a TN: true negative.

b FP: false positive.

c FN: false negative.

d TP: true positive.

e PPV: positive predictive value.

f NPV: negative predictive value.

g RF: random forest.

h XGBoost: extreme gradient boosting.

TabNet and TabTransformer yielded mixed results. Although TabNet achieved competitive metrics for Class 0, its macro-average *F*_1_-scores and overall performance were impacted by its frequently low recall for Class 1. TabTransformer showed moderate accuracy with higher variability across waves, though its Class 1 *F*_1_-scores remained lower than those of RF and XGBoost.

**Figure 1.**
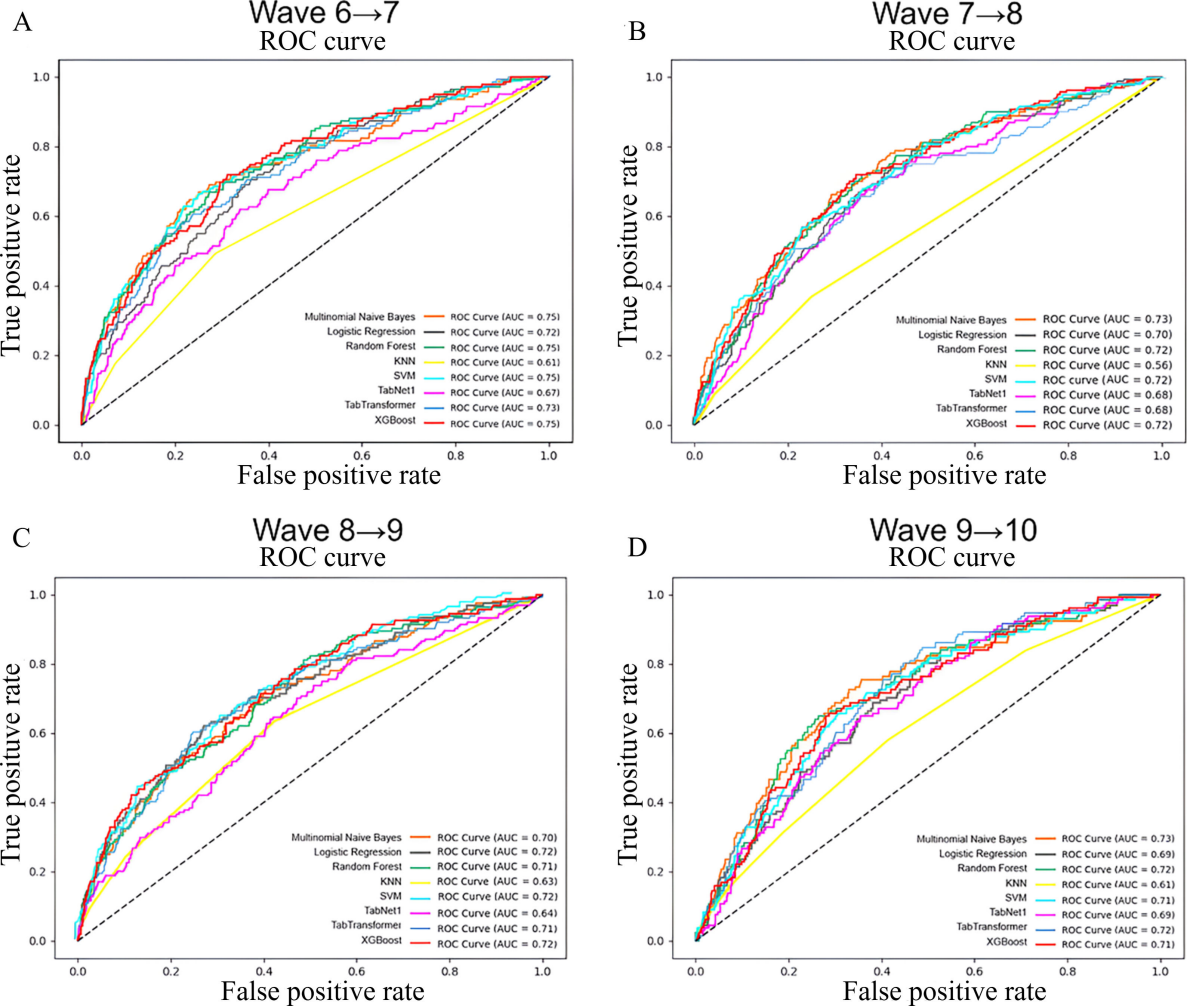
Receiver operating characteristic (ROC) curves and corresponding area under the receiver operating characteristic curve (AUROC) scores for machine learning models predicting depressive symptoms at follow-up in the English Longitudinal Study of Ageing (ELSA), waves 6‐10 (older adults aged ≥50 years). Panels show prediction from (A) wave 6→7, (B) wave 7→8, (C) wave 8→9, and (D) wave 9→10. Clinically significant depressive symptoms were defined using 8-item Center for Epidemiologic Studies Depression Scale (CES-D 8) score ≥4. AUC: area under the curve; KNN: k-nearest neighbors; ROC: receiver operating characteristic; SVM: support vector machine; XGBoost: extreme gradient boosting.

MNB emerged as a relatively balanced model, with acceptable *F*_1_-scores for both Class 0 and Class 1. As shown in Figure 1, its AUROC scores demonstrated consistent differentiation between the 2 classes, which makes it a viable choice for dealing with imbalanced datasets. KNN and LR were the weakest for Class 1, with *F*_1_-scores consistently below 0.35 and recall values often dropping below 0.40.

### Feature Importance Analysis

**Figure 2:**
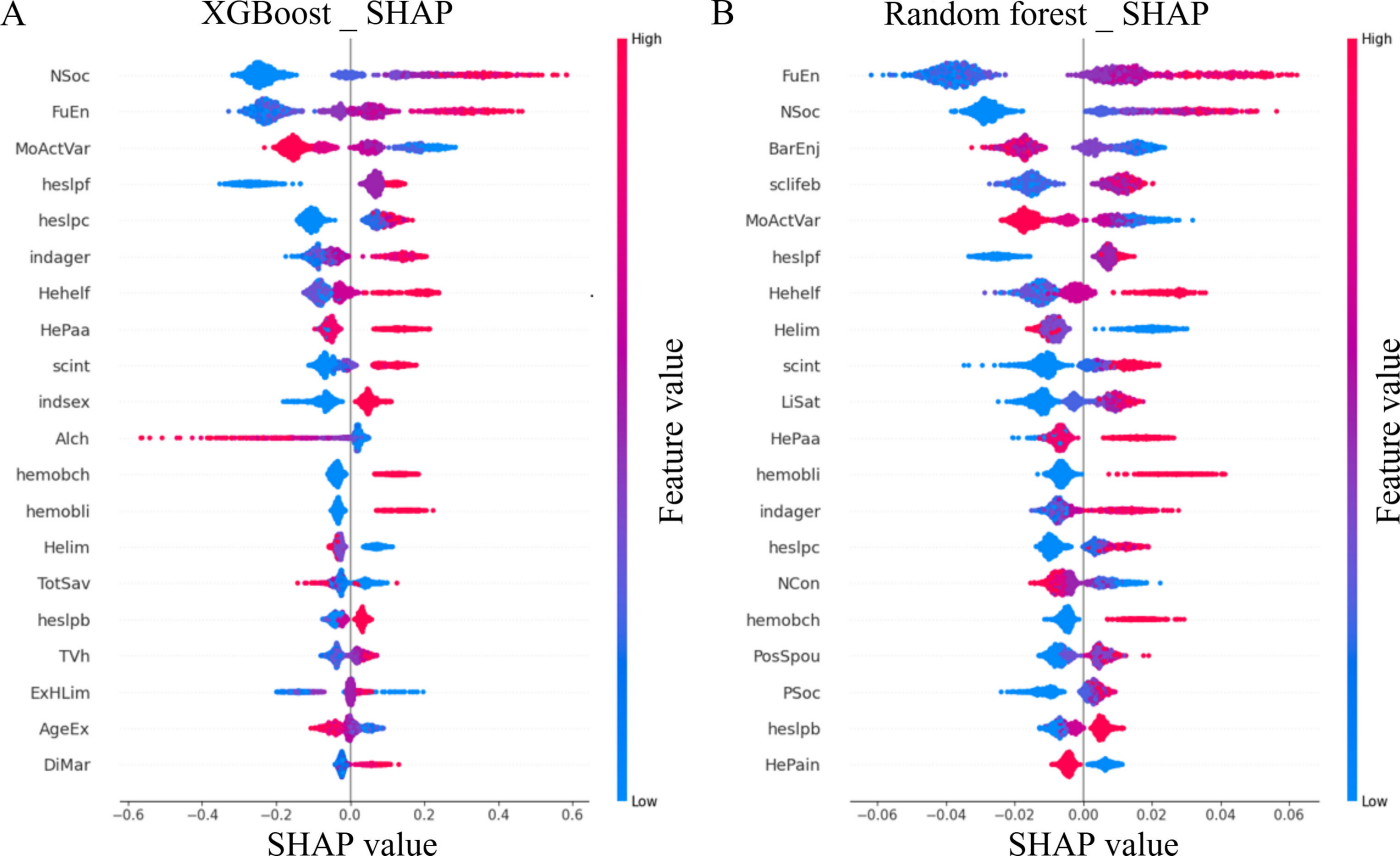
Shapley additive explanations (SHAP) feature importance for the top 20 predictors of depressive symptoms at follow-up using (A) extreme gradient boosting (XGBoost) and (B) random forest (RF) models among older adults (aged ≥50 years) in the English Longitudinal Study of Ageing (ELSA), wave 6. Each point represents an individual prediction. The x-axis shows SHAP values, which reveal each feature’s contribution to the model’s predicted probability of depressive symptoms at follow-up. Higher absolute SHAP values indicate greater predictive importance. The color gradient represents normalized feature values (blue=low and red=high).

As shown in Figure 2, SHAP values computed on the held-out test sets for the top-performing XGBoost and RF models highlighted several key predictors of depression. Common factors such as negative social interactions (NSoc), future outlook (FuEn), motivation for social and cultural activities (MoActVar), and overall sleep quality (heslpf) scored highly in both models, suggesting their importance in predicting depressive symptoms. Other features that are common across algorithms, such as self-reported general health (Hehelf) and frequency of internet use (scint), further emphasize their significance. These overlaps demonstrate the robustness and generalizability of these features in capturing multidimensional risk factors for depression. Some predictors overlap conceptually with CES-D items and may partly reflect symptom persistence; therefore, SHAP values should not be interpreted as evidence of independent causal effects. The SHAP analysis offers an interpretable framework for understanding model behavior and emphasizing the critical role of these common predictors in identifying individuals at risk for depression. Refer to Table 5 for a detailed breakdown of these features.

**Table 5.** Descriptions of top predictive features for depressive symptoms at follow-up identified through Shapley additive explanations (SHAP) feature importance analysis using random forest (RF) and extreme gradient boosting (XGBoost) models in the English Longitudinal Study of Ageing (ELSA), wave 6.

Features^a^	Feature description
heslpf	Sleep: rating sleep quality overall
indager	Definitive age variable collapsed at 90+ to avoid disclosure
NSoc	Frequency of negative social interactions and feelings of loneliness
FuEn	Frequency of positive future outlook and energy levels
NCon	Frequency of feelings of lack of control and social exclusion
BarEnj	Barriers preventing enjoyment: health, age, money, family responsibilities
AgeEx	Expectation of living to advanced ages (80 or 90+ y)
indsex	Definitive sex variable: priority disex, dhsex
Helim	Whether long-standing illness is limiting
HePaa	Severity of pain most of the time
hemobch	Mobility: difficulty getting up from a chair after sitting long periods
TVh	Total hours of TV watching during a week
heslpc	Sleep: number of days with trouble staying asleep in last month
MoActVar	Desire to engage more in cultural and social activities
ExHLim	Expectation (%) that health will limit ability to work before age 65
LTCEx	Expectation of needing to pay for long-term care
Hehelf	Self-reported general health
HeLWk	Self-reported health problem/disability that limits paid work
HePain	Whether often troubled with pain
hemobli	Mobility: difficulty lifting/carrying over 10 pounds
hemobwa	Mobility: difficulty walking 100 yards
scint	Frequency of internet or email use
PSoc	Frequency of positive social experiences and enjoyment
HeActb	Frequency of engaging in moderate sports/activities
Alch	Weighted alcohol consumption over the last 7 days
TotSav	Total financial wealth across all savings and assets
sclifeb	Agreement with the statement that life conditions are excellent
DiMar	Marital status of the respondent
Eyesight	Level of visual acuity
JobSat	Level of job satisfaction
LitMon	Level of financial limitations
DhWork	Whether in paid employment (last week)
LiSat	Level of life satisfaction and meaning
PosSpou	Level of positive spousal support
heslpb	Sleep: frequency of waking up several times at night
hemobcs	Mobility: difficulty climbing several flights of stairs
CaTNo	Number of activities respondent received help with in the last month
heslpa	Sleep: how often respondent has difficulty falling asleep

a Features are derived from self-completion questionnaires and computer-assisted personal interviewing, including demographic, physical health, and psychosocial domains. Feature names correspond to original English Longitudinal Study of Ageing variable codes.

**Figure 3.**
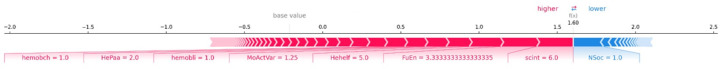
Shapley additive explanations (SHAP) force plot demonstrating individual-level feature contributions for a participant who developed clinically significant depressive symptoms at wave 7 (predicted risk: 85.7%).

Distinct risk profiles at the individual level are illustrated by SHAP force plots (Figures3  4). The participant who developed clinically significant depressive symptoms (predicted risk: 85.7%) exhibited convergence of multiple risk factors: mobility limitations (hemobch and hemobli), pain severity (HePaa), poor self-rated health (Hehelf), low future outlook and energy (FuEn), reduced desire to engage in activities (MoActVar), and digital exclusion (scint). In this case, red features pushed the prediction toward depression, whereas blue features pushed the prediction away from depression. Arrow width indicates the strength of each feature’s contribution. The base value represents the average model prediction across participants, and the final prediction for this individual was f(x)=1.60 in log-odds space. Low negative social interactions (NSoc) contributed in the protective direction, but the overall prediction remained high because multiple risk factors contributed more strongly toward depression. In contrast, the nondepressed participant (predicted risk: 7.8%) demonstrated strong protective factors, including low negative social interactions (NSoc), positive future outlook and energy (FuEn), good sleep quality (heslpf), adequate job recognition (JobRec), and diverse activity engagement (MoActVar). For this participant, the final prediction was f(x)=-2.48 in log-odds space, indicating very low depression risk, with minimal risk variables present. These plots illustrate how features contribute differently across individuals based on their specific risk profiles.

**Figure 4.**
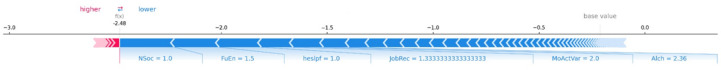
Shapley additive explanations (SHAP) force plot demonstrating individual-level feature contributions for a participant who remained nondepressed at wave 7 (predicted risk: 7.8%).

### Sensitivity Analyses

The results of the sensitivity analyses regarding CES-D 8 threshold selection and categorical encoding strategies are presented below.

#### Threshold Selection

The area under the curve (AUC) score, which measures the model’s capability to discriminate between participants who will and will not develop depression over 2 years, increased consistently with threshold severity: from 0.67 at threshold ≥3 (mild depression, 31.6% prevalence) to 0.75‐0.76 at threshold ≥4 (moderate, 14.2% prevalence) to 0.81‐0.82 at threshold ≥5 (severe, 8.0% prevalence; Table 6). This increase is based on the clinical reality that severe depression shows more significant and distinct symptoms, making it easier to distinguish from nondepressed individuals. In comparison, lower thresholds (≥3) capture subclinical presentations that overlap with nonclinical states, creating ambiguity and making discrimination more challenging.

**Table 6.** Comparative performance metrics of extreme gradient boosting (XGBoost) and random forest (RF) for predicting depressive symptoms at follow-up using different 8-item Center for Epidemiologic Studies Depression Scale (CES-D 8) thresholds (≥3, ≥4, and ≥5 symptoms) among older adults (aged ≥50 years) using the English Longitudinal Study of Ageing (ELSA), wave 6.

Model and threshold	Cases (n)^a^	Prevalence (%)^b^	AUC^c^^,^^d^	*F*_1_-score
XGBoost^e^				
≥3	1359	31.6	0.672	0.507
≥4	883	14.2	0.751	0.373
≥5	569	8.0	0.815	0.315
Random forest				
≥3	1359	31.6	0.669	0.495
≥4	883	14.2	0.756	0.373
≥5	569	8.0	0.813	0.311

a n indicates the number of participants who developed clinically significant depressive symptoms at wave 7 among those nondepressed at wave 6.

b Prevalence calculated as percentage of eligible participants meeting depression criteria at each threshold.

c AUC: area under the curve.

d AUC and *F*_1_-score show discriminative ability and balanced classification performance respectively. Both models show area under the receiver operating characteristic curve increases with threshold severity, while *F*_1_-score decreases due to prevalence-related trade-offs.

e XGBoost: extreme gradient boosting,

As shown in Table 6, the *F*_1_-score showed an inverse pattern, decreasing with higher thresholds. This decrease occurs mainly because of imbalanced classification tasks in which lower prevalence at stricter thresholds makes it challenging to maintain both high precision and high recall simultaneously.

Both models demonstrated nearly similar performance at each threshold, validating the consistency of these patterns. Feature importance also appeared to have stable patterns across all thresholds, with 3 main risk factors (self-rated health, future outlook/energy, and negative social interactions) revealed in the top 20 features for both models across all thresholds.

#### Categorical Encoding

Table 7 demonstrates the sensitivity analysis comparing ordinal and one-hot encoding across all 6 ML algorithms. Ordinal encoding achieved superior or equivalent performance in all algorithms. For tree-based models, ordinal encoding increased the AUC score by 0.03 for XGBoost and 0.02 for RF. The distance-based method KNN also benefited from ordinal encoding, showing a 0.02 AUC score increase. Linear models, LR, and SVM achieved equivalent AUC scores regardless of the chosen encoding strategy.

The *F*_1_-score increase in depressive symptoms prediction was more significant than the AUC score. XGBoost showed the highest improvement by 0.09, followed by RF and SVM. For MNB, ordinal encoding also achieved superior performance by 0.01 AUC score and by 0.03 *F*_1_-score increase. On average across all models, ordinal encoding improved the AUC score by 0.013 and the *F*_1_-score by 0.037, validating our feature engineering approach.

**Table 7.** Comparative performance metrics of 6 machine learning algorithms for predicting depressive symptoms at follow-up using different encoding strategies (ordinal and one-hot encoding) among older adults (aged ≥50 years) using the English Longitudinal Study of Ageing (ELSA), wave 6.

Model and encoding		AUC^a^	AUROC^b^ Δ^c^	*F*_1_-score	*F*_1_ Δ^d^
RF^e^			0.02		0.06
Ordinal		0.75		0.38	
One-Hot		0.73		0.32	
XGBoost^f^			0.03		0.09
Ordinal		0.75		0.33	
One-Hot		0.72		0.24	
KNN^g^			0.02		0
Ordinal		0.61		0.21	
One-Hot		0.59		0.21	
LR^h^			0		0
Ordinal		0.72		0.33	
One-Hot		0.72		0.33	
SVM^i^			0		0.04
Ordinal		0.75		0.39	
One-Hot		0.75		0.35	
MNB^j^			0.01		0.03
Ordinal		0.75		0.40	
One-Hot		0.74		0.37	

a AUC: area under the curve.

b AUROC: area under the receiver operating characteristic curve.

c AUROC Δ indicates the difference in AUC score (ordinal – one-hot).

d *F*_1_ Δ shows the difference in *F*_1_-score (ordinal – one-hot).

e RF: random forest.

f XGBoost: extreme gradient boosting.

g KNN: k-nearest neighbors.

h LR: logistic regression.

i SVM: support vector machine.

j MNB: multinomial naive Bayes.

### Clustering Analysis and Feature Representation

Clustering analysis was applied to explore if depression-related patterns could be identified beyond supervised performance. Features were grouped into 3 subsets, including demographic features, social engagement features, and physical health features, based on domain knowledge. Two clustering approaches were evaluated: (1) discretizing continuous data and applying ordinal encoding to categorical features, and (2) using TabTransformer embeddings for feature representation. TabTransformer embeddings were used to investigate whether a learned feature representation could better capture nonlinear relationships than simple encoding.

**Figure 5.**
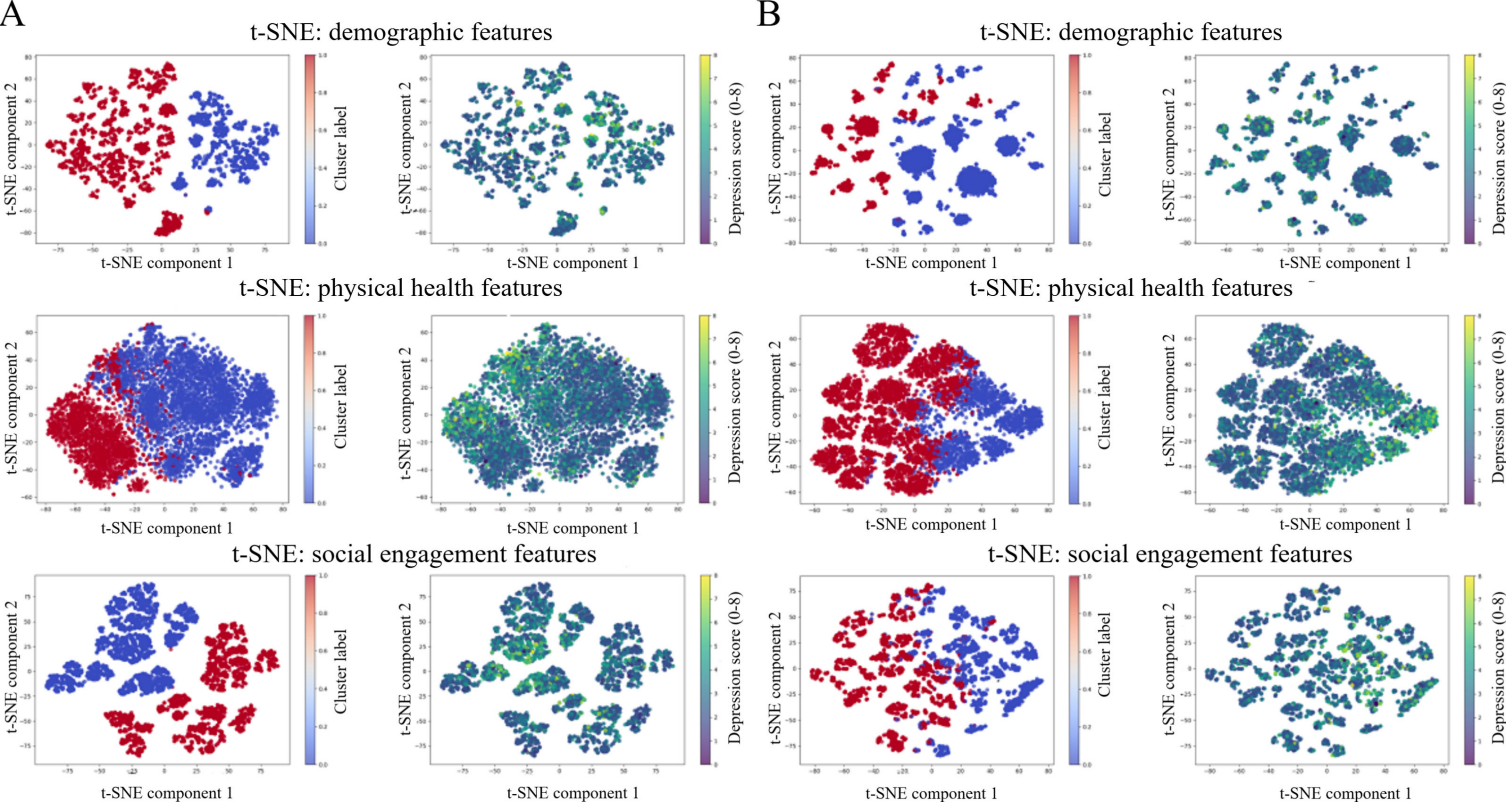
t-distributed stochastic neighbor embedding (t-SNE) visualizations and K-Means clustering results for older adults (aged ≥50 years) in the English Longitudinal Study of Ageing (ELSA), wave 6, using 2 different feature-representation strategies: (A) discretized and ordinally encoded features combining numerical and categorical variables and (B) TabTransformer embeddings integrating categorical and continuous features through attention-based representation learning. Each panel displays 3 feature subsets—demographic, physical health, and social engagement—used to explore latent groupings related to depression risk. Color bars show depression severity scores (8-item Center for Epidemiologic Studies Depression Scale [CES-D 8], right) and cluster labels (left, k=3).

Across both representations and all 3 subsets, the silhouette scores were consistently negative, indicating challenges in forming clear clusters. Accordingly, t-SNE visualizations were used for qualitative exploration rather than to demonstrate cluster separability. While qualitative inspection of the t-SNE visualizations (Figure 5) suggested informative exploratory patterns related to depression scores, these visual structures should be interpreted cautiously, as t-SNE can produce apparent groupings even when substantial overlap exists in the original feature space. Patterns were most visible in social engagement features, less visible in demographic features, and minimal in physical health features.

## Discussion

Using data from the nationally representative ELSA study, we developed ML models to predict clinically significant depressive symptoms at 2-year follow-up among adults aged 50 years and above. Our goal was to identify individuals at higher risk for developing elevated depressive symptoms and provide a foundation for potential early interventions.

Although statistical models are highly practical for hypothesis testing, they rely on linear assumptions and predefined interaction structures, which may limit their capability to capture the complex relationships underlying depressive symptoms at follow-up in older adults. In contrast, ML and deep learning approaches can model nonlinear interactions among psychological, social, and health-related domains, enabling a more comprehensive representation of depression risk factors. Applying explainability methods such as SHAP allows us to retain interpretability, despite models’ complexity.

In our study, traditional ML models, such as RF, SVM, and XGBoost, demonstrated better performance than deep learning techniques such as TabNet and TabTransformer. These results may be explained by the nature of our dataset, which contains mostly ordinal categorical features. Traditional ML models effectively handle ordinal data through straightforward encoding techniques that preserve the inherent order and relationships within the features. In contrast, TabNet and TabTransformer use complex embedding strategies designed to capture complex feature interactions. However, they may not fully leverage the ordinal characteristics of the data, leading to weaker performance. Furthermore, simpler models such as KNN and LR showed an insufficient ability to recognize complex patterns in the data, leading to lower predictive accuracy. This suggests that while simpler algorithms can perform well in some cases, their effectiveness may decline significantly when applied to complex, multidimensional phenomena like depression.

Although MNB has a probabilistic nature and assumes feature independence, it still yielded competitive results. This suggests that in certain contexts, when applying appropriate preprocessing steps, probabilistic models are capable of capturing depression risk factors. In our study to meet the model’s requirements, numerical variables were discretized into categorical bins. These findings reveal why understanding a dataset’s characteristics is important for selecting the best model. Choosing a model that is inherently aligned with the dataset’s characteristics can improve both performance and interpretability. Selecting the right model is even more important when dealing with datasets that contain a mix of categorical (nominal and ordinal) and numerical variables.

In contrast to earlier studies, our models achieved AUROC values comparable to or higher than those reported in prior longitudinal work, as RF and XGBoost both reached an AUROC of 0.72 [7,19,20]. However, direct comparisons of predictive performance should be interpreted cautiously considering the differences in cohorts, target definitions, and predictor sets. Zheng et al [20] reported an AUROC of 0.673, while Su et al [19] achieved an AUROC of 0.629 using LR with lasso regularization. In our study, we aimed to identify independent predictors of future depressive symptoms by excluding baseline depressive symptom status from the models to minimize data leakage and preserve predictor independence. In contrast, Song et al [7] incorporated baseline psychological scales as predictors and reported a higher AUROC of 0.791. Although this reflects stronger predictive performance, including baseline symptom measures makes interpretation more challenging, as predictions may partly capture symptom persistence rather than future risk. In our study, participants with clinically significant depressive symptoms at baseline were excluded, which minimizes the risk of directly capturing symptom persistence.

Our feature importance analysis identified significant predictors of depressive symptoms at follow-up, including negative social interactions, sleep quality, positive social and spousal support, self-rated health, and life satisfaction. These findings align with prior research. For example, Zheng et al [20] highlighted sex, self-rated health status, physical pain, and marital status as key predictors, consistent with our results, although occupation and eyesight were not emphasized in our analysis. Further supporting the role of functional health and social engagement in predicting depressive symptoms, Su et al [19] had emphasized the significance of self-rated health, activities of daily living, and marital status. Similarly, Song et al [7] pointed out that perceived health, social support, and life satisfaction are key indicators, emphasizing that depression spans physical, social, and emotional areas. Interestingly, they also found that nutritional risk and emotional instability are significant predictors, factors that we did not focus on in our analysis. These differences may arise from variations in study populations, data collection methods, or feature engineering approaches.

The SHAP force plots provide individual-level validation of the aggregate feature importance findings. Key predictors observed across both XGBoost and RF models, including negative social interactions, future outlook and energy, and activity motivation, emerge as the main factors influencing individual predictions in both cases examined. In the depressed participant, poor self-rated health, low future outlook, reduced activity motivation, and digital exclusion collectively push the prediction toward depression, while in the nondepressed participant, low negative social interactions, positive future outlook, good sleep quality, and varied activity engagement provide strong protective effects. This consistency between population-level feature importance and individual-level explanations improves confidence in the clinical relevance of these predictors and implies that the found risk factors operate meaningfully at both aggregate and individual levels.

The sensitivity analysis conducted demonstrated strong consistency between XGBoost and RF across all thresholds, suggesting that results represent a true clinical situation rather than model-specific artifacts. It is important to consider that CES-D 8 is an abbreviated version of the original 20-item scale. Therefore, a one-point difference in threshold implies a significant change in depression severity. This explains the notable performance variation observed: the AUC score increases as the threshold becomes higher, mainly due to the improved ability of the model to detect well-defined cases with obvious symptom profiles. Conversely, *F*_1_-scores decrease with severity, since this parameter is heavily influenced by prevalence. As the threshold increases, the imbalance degree of the dataset increases, which makes it more challenging to maintain both high precision and recall. The main risk factors, self-rated health, psychological well-being, negative social interactions, remained stable across thresholds in both models, implying these predictors are important regardless of the threshold chosen.

In addition to threshold selection, we conducted sensitivity analysis to examine categorical encoding strategies. The consistent outperformance of ordinal encoding over one-hot encoding supports the suitability of our feature engineering strategy. Ordinal encoding preserves natural ordering of many ELSA variables, such as self-rated health, mobility limitations, and pain severity, while one-hot encoding discards it. It was especially important for tree-based models, such as XGBoost and RF, and distance-based methods, such as KNN, which directly benefit from preserved ordinal relationships. The improved *F*_1_-scores with ordinal encoding lead to better identification of at-risk individuals for early intervention.

In order to address high dimensionality and missing data, some recent works have integrated dimensionality reduction into deep learning architectures. Tutsoy and Sumbul [31], for instance, suggested dimensionality reduction based on correlation and target similarity for the diagnosis of thyroid cancer. This method reduced 39-dimensional biomarker data to 5‐10 features while handling up to 88% random missingness and achieving 83% testing accuracy. However, these methods have limitations when applied to epidemiological cohorts with diverse feature types and are best suited to continuous biomarker data. We focus on encoding-aware preprocessing for longitudinal cohort data like ELSA because, in particular, categorical variables need to be encoded before correlation analysis, since correlation measures might not be directly comparable across categorical and continuous data types, and standard correlation does not respect ordinal structure.

Building on the insights from feature importance analysis, clustering provided an additional layer of understanding. Despite negative Silhouette scores across all subsets, highlighting the inherent difficulty of clustering high-dimensional data, t-SNE visualizations revealed depression-related patterns. Among the subsets, social engagement features exhibited the clearest groupings, emphasizing the critical role of social factors such as social support, which were identified as key predictors in our feature importance analysis. While physical health features showed less clear clustering, reflecting their more indirect relationship with depression severity, demographic features showed moderate separation, matching predictors such as self-rated health and marital status.

Our study has several limitations that should be acknowledged. The use of self-reported questionnaires may introduce reporting bias, as participants may underreport or overreport symptoms and behaviors, potentially affecting the reliability of some predictors. Additionally, depressive symptoms in this study were assessed using the CES-D 8 scale, a widely used screening tool with strong psychometric properties; however, it is not a clinical diagnostic tool and may lead to misclassification, particularly among individuals with subthreshold symptoms. Some baseline predictors, such as sleep quality and physical functioning, may overlap conceptually with CES-D items, which could result in the models partly capturing symptom persistence rather than entirely new symptom emergence. To minimize direct information leakage, participants exceeding the CES-D threshold at baseline were excluded, and all predictors were measured prior to the follow-up outcome. Excluding individuals with severe psychiatric conditions may also limit the ability to generalize the results to high-risk populations. Finally, negative silhouette scores indicate that depression risk in older adults may vary gradually rather than forming clear, distinct groups. Apparent groupings observed in t-SNE visualizations should therefore be interpreted cautiously.

There are several sources of uncertainty in model predictions. Sampling uncertainty arises from missingness associated with older age, lower education, and baseline depression, potentially violating missing-at-random assumptions. Methodological uncertainty results from modeling choices such as threshold selection, hyperparameter tuning, and encoding. Feature-related uncertainty relates to both feature reduction (6100 to 120 variables) and unmeasured confounding such as genetic vulnerability or medication use. The 2-year prediction interval introduces temporal uncertainty, as major life events may occur between baseline and follow-up assessments.

To assess the real-world application of the model, further study should validate the predictive model’s performance using a clinical dataset such as general practitioner records. The predictive analysis in this study relied on single-wave data to forecast depressive symptoms at the subsequent wave, which may not fully capture longer-term temporal dynamics of depression; incorporating information from multiple previous waves could provide a more comprehensive understanding of depression trajectories.

Despite these limitations, our findings demonstrate that ML algorithms using the ELSA dataset are feasible in predicting depressive symptoms at 2-year follow-up in the aging population. The results suggested that incorporating a wide range of features from different domains can enhance the model’s prediction capability. Notably, traditional ML models, such as SVM, RF, MNB, and XGBoost, performed better than deep learning techniques, emphasizing the value of aligning model selection and preprocessing with the properties of the dataset. These findings contribute to a deeper understanding of depression risk and support the development of early intervention strategies and scalable risk stratification approaches for aging populations.

## Supplementary material

10.2196/84744 Multimedia Appendix 1 Supplementary tables of baseline sociodemographic characteristics and model performance metrics by depression status across English Longitudinal Study of Ageing (ELSA) waves 7-9.

## Abbreviations

AUC: area under the curve
AUROC: area under the receiver operating characteristic curve
CES-D: Center for Epidemiologic Studies Depression Scale
CES-D 8: 8-item Center for Epidemiologic Studies Depression Scale
ELSA: English Longitudinal Study of Ageing
KNN: k-nearest neighbors
LR: logistic regression
ML: machine learning
MNB: multinomial naive Bayes
RF: random forest
SHAP: Shapley additive explanations
SVM: support vector machine
t-SNE: t-distributed stochastic neighbor embedding
XGBoost: extreme gradient boosting
