# Supplementary material for: Prediction of Clinically Significant Depressive Symptoms at 2-Year Follow-Up in Older Adults: Machine Learning Study Using the English Longitudinal Study of Ageing
Source: JMIR Form Res. 2026 Jul 2;10:e84744. doi: 10.2196/84744 (PMC13325927; doi:10.2196/84744)
Supplement: Multimedia Appendix 1 [file formative-v10-e84744-s001.docx]

# Supplementary Material

The following tables provide baseline sociodemographic characteristics (including gender, age group, marital status, and cultural back- ground) for participants included in ELSA Waves 7, 8, and 9, grouped by depression status.

**Table S1.** Sociodemographic characteristics by levels of depression among participants in Wave 7

| Variable | All participants (n=7980) | Non-depressed (n=6159) | Depressed (n=1821) |
| --- | --- | --- | --- |

| **Gender**  Male Female | 3586 (45%)  4394 (55%) | 2964 (48%)  3195 (52%) | 622 (34%)  1199 (66%) |
| --- | --- | --- | --- |
| **Age**  Age ≥ 65  Age *<* 65 | 4745 (59%)  3235 (41%) | 3526 (57%)  2633 (43%) | 1219 (67%)  602 (33%) |
| **Marital Status**  Single (Never married) Married/Civil Partnership  Previously Married (Separated/Divorced/Widowed) N/A | 495 (6.2%)  5359 (67.2%)  2124 (26.6%)  2 (∼0%) | 394 (6.4%)  4374 (71%)  1389 (22.6%)  2 (∼0%) | 101 (5.5%)  985 (54.1%)  735 (40.4%)  0 (0%) |
| **Cultural Background**  English Irish/Scottish/Welsh Other European  Other cultural background N/A | 6652 (83.3%)  421 (5.3%)  53 (0.7%)  168 (2.1%)  686 (8.6%) | 5143 (83.5%)  334 (5.4%)  45 (0.7%)  143 (2.3%)  494 (8%) | 1509 (82.9%)  87 (4.8%)  8 (0.4%)  25 (1.4%)  192 (10.5%) |

**Table S2.** Sociodemographic characteristics by levels of depression among participants in Wave 8

| Variable | All participants (n=7809) | Non-depressed (n=5749) | Depressed (n=2060) |
| --- | --- | --- | --- |

| **Gender**  Male Female | 3428 (44%)  4381 (56%) | 2713 (47%)  3036 (53%) | 715 (35%)  1345 (65%) |
| --- | --- | --- | --- |
| **Age**  Age ≥ 65  Age *<* 65 | 5074 (65%)  2735 (35%) | 3683 (64%)  2066 (36%) | 1391 (68%)  669 (32%) |
| **Marital Status**  Single (Never married) Married/Civil Partnership  Previously Married (Separated/Divorced/Widowed) N/A | 508 (6.5%)  5212 (66.8%)  2085 (26.7%)  4 (∼0%) | 361 (6.3%)  4068 (70.8%)  1318 (22.9%)  2 (∼0%) | 147 (7.1%)  1144 (55.5%)  767 (27.2%)  2 (0%) |

**Table S3.** Sociodemographic characteristics by levels of depression among participants in Wave 9

| Variable | All participants (n=7869) | Non-depressed (n=5891) | Depressed (n=1978) |
| --- | --- | --- | --- |

| **Gender**  Male Female | 3417 (43%)  4452 (57%) | 2739 (46%)  3152 (54%) | 678 (34%)  1300 (66%) |
| --- | --- | --- | --- |
| **Age**  Age ≥ 65  Age *<* 65 | 5000 (64%)  2869 (36%) | 3662 (62%)  2229 (38%) | 1338 (68%)  640 (32%) |
| **Marital Status**  Single (Never married) Married/Civil Partnership  Previously Married (Separated/Divorced/Widowed) N/A | 611 (7.8%)  5239 (66.6%)  2018 (25.6%)  1 (∼0%) | 457 (7.7%)  4134 (70.2%)  1299 (22.1%)  1 (∼0%) | 154 (7.8%)  1105 (55.9%)  719 (36.3%)  0 (0%) |

Table S4 shows the mean performance metrics for Class 0 across all waves. Detailed wave-specific results for each wave (Class 1) are presented in Tables S5 to S8, and the corresponding metrics for the non-depressed class (Class 0) are provided in Tables S9 to S12.

**Table S4.** Performance Metrics for Predicting the Non-Depressed Class (Class 0) Across Waves (Mean ± Std)

| Model | Precision (Class 0) | Recall (Class 0) | F1-Score (Class 0) |
| --- | --- | --- | --- |

| RF | 0.90 ± 0.02 | 0.80 ± 0.04 | 0.85 ± 0.03 |
| --- | --- | --- | --- |
| TabNet | 0.89 ± 0.02 | 0.78 ± 0.09 | 0.83 ± 0.05 |
| XGBoost | 0.90 ± 0.01 | 0.75 ± 0.07 | 0.82 ± 0.04 |
| KNN | 0.87 ± 0.02 | 0.85 ± 0.08 | 0.86 ± 0.05 |
| LR | 0.89 ± 0.02 | 0.82 ± 0.04 | 0.85 ± 0.02 |
| SVM | 0.91 ± 0.02 | 0.76 ± 0.07 | 0.82 ± 0.04 |
| MNB | 0.91 ± 0.02 | 0.78 ± 0.03 | 0.84 ± 0.02 |
| TabTransformer | 0.91 ± 0.01 | 0.73 ± 0.09 | 0.81 ± 0.06 |

**Table S5.** Performance metrics for predicting depression onset in Wave 6 (Class 1). Values are mean ± standard deviation over five runs with different random seeds.

| **Model** | **Precision (C1)** | **Recall (C1)** | **F1-score (C1)** | **Accuracy** | **Macro Avg F1** | **AUC** |
| --- | --- | --- | --- | --- | --- | --- |

| RF | 0.30 ± 0.02 | 0.52 ± 0.05 | 0.38 ± 0.03 | 0.79 ± 0.01 | 0.63 ± 0.02 | 0.75 ± 0.02 |
| --- | --- | --- | --- | --- | --- | --- |
| TabNet | 0.26 ± 0.03 | 0.39 ± 0.07 | 0.31 ± 0.05 | 0.79 ± 0.02 | 0.59 ± 0.04 | 0.67 ± 0.04 |
| XGBoost | 0.30 ± 0.02 | 0.51 ± 0.04 | 0.38 ± 0.03 | 0.79 ± 0.01 | 0.63 ± 0.02 | 0.75 ± 0.02 |
| KNN | 0.26 ± 0.04 | 0.18 ± 0.06 | 0.21 ± 0.06 | 0.84 ± 0.02 | 0.56 ± 0.05 | 0.61 ± 0.05 |
| LR | 0.28 ± 0.02 | 0.42 ± 0.05 | 0.33 ± 0.03 | 0.79 ± 0.01 | 0.61 ± 0.02 | 0.72 ± 0.03 |
| SVM | 0.29 ± 0.02 | 0.56 ± 0.05 | 0.39 ± 0.03 | 0.78 ± 0.01 | 0.63 ± 0.02 | 0.75 ± 0.02 |
| MNB | 0.30 ± 0.02 | 0.58 ± 0.04 | 0.40 ± 0.03 | 0.78 ± 0.01 | 0.63 ± 0.02 | 0.75 ± 0.02 |
| TabTransformer | 0.30 ± 0.02 | 0.55 ± 0.05 | 0.39 ± 0.04 | 0.78 ± 0.01 | 0.63 ± 0.03 | 0.73 ± 0.03 |

**Table S6.** Performance metrics for predicting depression onset in Wave 7 (Class 1). Values are mean ± standard deviation over five runs with different random seeds.

| **Model** | **Precision (C1)** | **Recall (C1)** | **F1-score (C1)** | **Accuracy** | **Macro Avg F1** | **AUC** |
| --- | --- | --- | --- | --- | --- | --- |

| RF | 0.32 ± 0.03 | 0.45 ± 0.06 | 0.37 ± 0.04 | 0.77 ± 0.02 | 0.61 ± 0.03 | 0.72 ± 0.02 |
| --- | --- | --- | --- | --- | --- | --- |
| TabNet | 0.27 ± 0.04 | 0.48 ± 0.07 | 0.35 ± 0.05 | 0.72 ± 0.03 | 0.59 ± 0.04 | 0.68 ± 0.04 |
| XGBoost | 0.30 ± 0.03 | 0.53 ± 0.06 | 0.39 ± 0.04 | 0.74 ± 0.02 | 0.61 ± 0.03 | 0.72 ± 0.03 |
| KNN | 0.21 ± 0.05 | 0.37 ± 0.08 | 0.27 ± 0.06 | 0.69 ± 0.03 | 0.54 ± 0.05 | 0.56 ± 0.05 |
| LR | 0.28 ± 0.03 | 0.41 ± 0.06 | 0.33 ± 0.04 | 0.75 ± 0.02 | 0.59 ± 0.03 | 0.70 ± 0.03 |
| SVM | 0.28 ± 0.03 | 0.61 ± 0.07 | 0.38 ± 0.05 | 0.70 ± 0.03 | 0.59 ± 0.04 | 0.72 ± 0.03 |
| MNB | 0.30 ± 0.03 | 0.56 ± 0.06 | 0.39 ± 0.04 | 0.74 ± 0.02 | 0.61 ± 0.03 | 0.73 ± 0.03 |
| TabTransformer | 0.27 ± 0.04 | 0.54 ± 0.07 | 0.36 ± 0.05 | 0.71 ± 0.03 | 0.59 ± 0.04 | 0.68 ± 0.04 |

**Table S7.** Performance metrics for predicting depression onset in Wave 8 (Class 1). Values are mean ± standard deviation over five runs with different random seeds.

| **Model** | **Precision (C1)** | **Recall (C1)** | **F1-score (C1)** | **Accuracy** | **Macro Avg F1** | **AUC** |
| --- | --- | --- | --- | --- | --- | --- |

| RF | 0.33 ± 0.03 | 0.49 ± 0.05 | 0.40 ± 0.04 | 0.75 ± 0.02 | 0.62 ± 0.03 | 0.71 ± 0.04 |
| --- | --- | --- | --- | --- | --- | --- |
| TabNet | 0.29 ± 0.05 | 0.30 ± 0.07 | 0.30 ± 0.06 | 0.76 ± 0.03 | 0.58 ± 0.04 | 0.64 ± 0.05 |
| XGBoost | 0.29 ± 0.04 | 0.56 ± 0.06 | 0.38 ± 0.05 | 0.70 ± 0.03 | 0.59 ± 0.03 | 0.72 ± 0.04 |
| KNN | 0.32 ± 0.06 | 0.24 ± 0.08 | 0.28 ± 0.07 | 0.79 ± 0.04 | 0.58 ± 0.05 | 0.63 ± 0.06 |
| LR | 0.32 ± 0.03 | 0.55 ± 0.07 | 0.40 ± 0.05 | 0.73 ± 0.03 | 0.61 ± 0.04 | 0.72 ± 0.03 |
| SVM | 0.35 ± 0.04 | 0.47 ± 0.05 | 0.40 ± 0.04 | 0.77 ± 0.02 | 0.63 ± 0.03 | 0.72 ± 0.04 |
| MNB | 0.30 ± 0.03 | 0.54 ± 0.06 | 0.39 ± 0.04 | 0.72 ± 0.03 | 0.60 ± 0.03 | 0.70 ± 0.04 |
| TabTransformer | 0.32 ± 0.04 | 0.60 ± 0.07 | 0.42 ± 0.05 | 0.73 ± 0.03 | 0.62 ± 0.04 | 0.71 ± 0.03 |

**Table S8.** Performance metrics for predicting depression onset in Wave 9 (Class 1). Values are mean ± standard deviation over five runs with different random seeds.

| **Model** | **Precision (C1)** | **Recall (C1)** | **F1-score (C1)** | **Accuracy** | **Macro Avg F1** | **AUC** |
| --- | --- | --- | --- | --- | --- | --- |

| RF | 0.34 ± 0.02 | 0.64 ± 0.04 | 0.44 ± 0.03 | 0.72 ± 0.02 | 0.63 ± 0.02 | 0.72 ± 0.03 |
| --- | --- | --- | --- | --- | --- | --- |
| TabNet | 0.27 ± 0.03 | 0.63 ± 0.05 | 0.38 ± 0.03 | 0.65 ± 0.03 | 0.57 ± 0.03 | 0.69 ± 0.03 |
| XGBoost | 0.30 ± 0.02 | 0.69 ± 0.04 | 0.41 ± 0.03 | 0.67 ± 0.03 | 0.59 ± 0.02 | 0.71 ± 0.02 |
| KNN | 0.25 ± 0.03 | 0.31 ± 0.05 | 0.28 ± 0.04 | 0.73 ± 0.02 | 0.56 ± 0.03 | 0.61 ± 0.03 |
| LR | 0.31 ± 0.02 | 0.37 ± 0.04 | 0.34 ± 0.03 | 0.75 ± 0.02 | 0.59 ± 0.02 | 0.69 ± 0.02 |
| SVM | 0.30 ± 0.02 | 0.66 ± 0.04 | 0.41 ± 0.03 | 0.68 ± 0.03 | 0.59 ± 0.02 | 0.71 ± 0.02 |
| MNB | 0.36 ± 0.02 | 0.56 ± 0.04 | 0.44 ± 0.03 | 0.75 ± 0.02 | 0.64 ± 0.02 | 0.73 ± 0.02 |
| TabTransformer | 0.28 ± 0.03 | 0.72 ± 0.05 | 0.40 ± 0.03 | 0.63 ± 0.03 | 0.57 ± 0.03 | 0.72 ± 0.03 |

**Table S9.** Performance Metrics for Predicting the Non-Depressed in Wave 6 (Class 0). Values are mean ± standard deviation over five runs with different random seeds.

| **Model** | **Precision (C0)** | **Recall (C0)** | **F1-score (C0)** |
| --- | --- | --- | --- |

| RF | 0.92 ± 0.05 | 0.83 ± 0.04 | 0.88 ± 0.06 |
| --- | --- | --- | --- |
| TabNet | 0.91 ± 0.03 | 0.84 ± 0.07 | 0.87 ± 0.05 |
| XGBoost | 0.92 ± 0.06 | 0.83 ± 0.05 | 0.87 ± 0.07 |
| KNN | 0.89 ± 0.04 | 0.93 ± 0.02 | 0.91 ± 0.03 |
| LR | 0.91 ± 0.02 | 0.85 ± 0.06 | 0.88 ± 0.05 |
| SVM | 0.93 ± 0.07 | 0.81 ± 0.08 | 0.86 ± 0.06 |
| MNB | 0.93 ± 0.04 | 0.81 ± 0.05 | 0.87 ± 0.04 |
| TabTransformer | 0.93 ± 0.06 | 0.82 ± 0.03 | 0.87 ± 0.05 |

**Table S10.** Performance Metrics for Predicting the Non-Depressed in Wave 7 (Class 0). Values are mean ± standard deviation over five runs with different random seeds.

| **Model** | **Precision (C0)** | **Recall (C0)** | **F1-score (C0)** |
| --- | --- | --- | --- |

| RF | 0.89 ± 0.05 | 0.82 ± 0.04 | 0.86 ± 0.06 |
| --- | --- | --- | --- |
| TabNet | 0.89 ± 0.03 | 0.77 ± 0.06 | 0.82 ± 0.04 |
| XGBoost | 0.90 ± 0.06 | 0.78 ± 0.05 | 0.84 ± 0.05 |
| KNN | 0.87 ± 0.04 | 0.75 ± 0.07 | 0.80 ± 0.06 |
| LR | 0.88 ± 0.02 | 0.82 ± 0.04 | 0.85 ± 0.03 |
| SVM | 0.91 ± 0.07 | 0.72 ± 0.08 | 0.80 ± 0.07 |
| MNB | 0.91 ± 0.05 | 0.77 ± 0.06 | 0.83 ± 0.05 |
| TabTransformer | 0.90 ± 0.06 | 0.74 ± 0.05 | 0.81 ± 0.04 |

**Table S11.** Performance Metrics for Predicting the Non-Depressed in Wave 8 (Class 0). Values are mean ± standard deviation over five runs with different random seeds.

| **Model** | **Precision (C0)** | **Recall (C0)** | **F1-score (C0)** |
| --- | --- | --- | --- |

| RF | 0.89 ± 0.04 | 0.80 ± 0.05 | 0.84 ± 0.04 |
| --- | --- | --- | --- |
| TabNet | 0.86 ± 0.06 | 0.85 ± 0.04 | 0.86 ± 0.05 |
| XGBoost | 0.89 ± 0.05 | 0.73 ± 0.06 | 0.80 ± 0.05 |
| KNN | 0.86 ± 0.03 | 0.90 ± 0.04 | 0.88 ± 0.04 |
| LR | 0.90 ± 0.02 | 0.76 ± 0.05 | 0.82 ± 0.03 |
| SVM | 0.89 ± 0.05 | 0.83 ± 0.04 | 0.86 ± 0.05 |
| MNB | 0.89 ± 0.04 | 0.75 ± 0.05 | 0.82 ± 0.04 |
| TabTransformer | 0.91 ± 0.03 | 0.75 ± 0.06 | 0.82 ± 0.04 |

**Table S12.** Performance Metrics for Predicting the Non-Depressed in Wave 9 (Class 0). Values are mean ± standard deviation over five runs with different random seeds.

| **Model** | **Precision (C0)** | **Recall (C0)** | **F1-score (C0)** |
| --- | --- | --- | --- |

| RF | 0.91 ± 0.03 | 0.74 ± 0.06 | 0.82 ± 0.04 |
| --- | --- | --- | --- |
| TabNet | 0.90 ± 0.05 | 0.65 ± 0.07 | 0.76 ± 0.05 |
| XGBoost | 0.91 ± 0.04 | 0.66 ± 0.05 | 0.77 ± 0.04 |
| KNN | 0.85 ± 0.06 | 0.81 ± 0.04 | 0.83 ± 0.05 |
| LR | 0.87 ± 0.03 | 0.83 ± 0.05 | 0.85 ± 0.04 |
| SVM | 0.91 ± 0.04 | 0.68 ± 0.06 | 0.78 ± 0.05 |
| MNB | 0.90 ± 0.05 | 0.79 ± 0.04 | 0.84 ± 0.05 |
| TabTransformer | 0.91 ± 0.03 | 0.61 ± 0.07 | 0.73 ± 0.05 |
